# Associations of Combined Transcranial Magnetic Stimulation and Transcranial Direct Current Stimulation With Psychological and Functional Outcomes After Stroke: A Retrospective Cohort Study

**DOI:** 10.62641/aep.2337

**Published:** 2026-10-15

**Authors:** Yuehao Wu, Yusha Shen, Meng Huang

**Affiliations:** ^1^Department of Neurology, The First People’s Hospital of Linping District, 311100 Hangzhou, Zhejiang, China

**Keywords:** stroke, neuromodulation therapy, TMS, tDCS, Fugl-Meyer Assessment, post-stroke rehabilitation

## Abstract

**Background:**

To examine whether combined transcranial magnetic stimulation (TMS) and transcranial direct current stimulation (tDCS), delivered with standard rehabilitation, was associated with post-stroke depression and anxiety symptoms and secondary neurological and motor outcomes.

**Methods:**

This retrospective cohort study included 180 patients classified according to treatment received in routine clinical practice: combined TMS + tDCS + standard rehabilitation (*n* = 60), standard rehabilitation alone (*n* = 60), TMS + standard rehabilitation (*n* = 30), or tDCS + standard rehabilitation (*n* = 30). Treatment selection reflected physician recommendations and patient preference. Beck Depression Inventory (BDI) and Generalized Anxiety Disorder 7-item scale (GAD-7) scores were primary outcomes; National Institutes of Health Stroke Scale (NIHSS), modified Rankin Scale (mRS), and Fugl-Meyer Assessment (FMA) scores were secondary outcomes. Outcomes were assessed at baseline, 4 weeks, and 8 weeks. Generalized estimating equations adjusted for prespecified measured covariates assessed group, time, and group × time associations. Dunnett-adjusted comparisons used standard rehabilitation as the reference.

**Results:**

No statistically significant differences were detected in the measured baseline characteristics (all *p* > 0.05). At 8 weeks, the combined group had lower median BDI scores [8.0 (6.0, 10.0) vs. 14.5 (11.5, 17.0)] and GAD-7 scores [6.0 (4.0, 8.0) vs. 9.0 (7.0, 11.0)] than the Standard Rehabilitation Group (both Dunnett-adjusted *p* < 0.001). Significant group × time associations were identified for BDI, GAD-7, NIHSS, mRS, and FMA outcomes (all *p* < 0.05). The combined group also had lower NIHSS and mRS scores and higher FMA scores than the standard rehabilitation group at 4 and 8 weeks. No severe adverse events were documented. Total recorded adverse-event rates were 8.3%, 5.0%, 3.3%, and 3.3% across the four groups, respectively, with no significant between-group difference (Fisher’s exact *p* = 0.584).

**Conclusions:**

In this retrospective cohort, combined TMS and tDCS with standard rehabilitation was associated with lower depression and anxiety symptom scores and more favorable neurological and motor outcome trajectories than standard rehabilitation. Non-random treatment allocation and residual confounding preclude causal interpretation; prospective randomized studies are required.

## Introduction

As a global public health crisis, stroke stands as the second most common cause of death and the primary driver of enduring disability [1, 2]. Each year, over 15 million people experience a stroke, with approximately 5 million surviving with permanent disability and another 5 million dying from the condition [3]. For survivors, the consequences of stroke involve both physical and psychological impairments, and post-stroke depression and anxiety have become the most common and harmful psychiatric outcomes, with a prevalence of 30%–50% in post-stroke patients [4]. Clinically, post-stroke depression and anxiety are not merely secondary emotional reactions; they are the strongest independent predictors of rehabilitation non-compliance, delayed motor recovery, and long-term functional decline [5]. Patients with elevated depressive or anxiety symptoms show significantly lower participation rates in rehabilitation training (reduced by approximately 40%–50%) [6], which directly translates into poorer functional independence outcomes [7]. Therefore, prioritizing the assessment and treatment of post-stroke psychiatric disturbances as primary therapeutic targets is not only clinically meaningful but also strategically essential: by establishing a baseline of psychological readiness, the efficacy of subsequent physical rehabilitation can be substantially amplified. These emotional disturbances not only directly reduce the quality of life of patients, but also significantly reduce rehabilitation compliance, inhibit the recovery of motor and neurological function, and even increase the risk of stroke recurrence and mortality [8]. In contrast, motor dysfunction (affecting 70–80% of patients) and neurological deficit are secondary physical impairments, which are also closely related to psychological state—patients with severe depression and anxiety often show slower motor function recovery [9]. However, current post-stroke rehabilitation strategies mostly focus on physical impairment improvement (e.g., motor function training), while ignoring the diagnosis and treatment of depression and anxiety, leading to unsatisfactory overall rehabilitation effects. These physical and psychological challenges not only bring heavy mental and economic burdens to individuals and families, but also impose significant pressure on healthcare systems worldwide.

In recent decades, advances in acute stroke care—such as intravenous thrombolysis and mechanical thrombectomy—have improved survival rates by restoring blood flow to the ischemic brain tissue [10, 11]. However, these treatments primarily target the acute phase and do not address the long-term neural repair and functional recovery needed to reduce disability. Typical post-stroke care strategies involving physical, occupational, and speech therapies, rely on repetitive motor training and task-specific practice to promote neural plasticity [12]. While these approaches can yield modest improvements, their efficacy is often limited, particularly in patients with moderate-to-severe impairments or those in the chronic post-stroke phase (more than 6 months after onset) [13]. Many patients remain unable to perform basic activities of daily living (ADL), such as dressing, feeding, or walking, even after prolonged rehabilitation, highlighting the need for more effective adjunctive therapies.

Neuromodulation therapy has emerged as a promising non-invasive approach to address this unmet need. Unlike traditional rehabilitation, which focuses on external motor training, neuromodulation directly targets the brain’s neural circuits to enhance plasticity and reorganize damaged networks [14]. Transcranial magnetic stimulation (TMS) and transcranial direct current stimulation (tDCS) are highly studied neuromodulation tools [15]. TMS uses pulsed magnetic fields to induce electrical currents in the cerebral cortex, modulating the excitability of specific brain regions—such as the primary motor cortex (M1)—that are critical for motor control [16]. tDCS employs a low-strength direct current (1-2 mA) via surface electrodes, altering neuron resting potential to raise excitability (anodal) or lower it (cathodal) [17]. Both techniques are non-invasive, well-tolerated, and have been shown in preclinical studies to promote synaptic strengthening, increase the expression of plasticity-related proteins (e.g., brain-derived neurotrophic factor, BDNF), and facilitate the reorganization of neural networks after stroke [18, 19].

Despite growing interest in neuromodulation for post-stroke rehabilitation, clinical evidence regarding its efficacy in alleviating post-stroke depression and anxiety remains inconsistent. Most earlier studies took motor or neurological function as the primary outcome, only set depression and anxiety as secondary indicators, and even lacked relevant assessment indicators. In addition, the limitations of previous studies with small sample sizes (fewer than 50 patients), short follow-up periods (less than 4 weeks), and single-modality neuromodulation treatment also make it difficult to draw definitive conclusions about the effect of neuromodulation on post-stroke emotional disorders. Meanwhile, the efficacy of combined TMS + tDCS in improving post-stroke depression and anxiety has not been fully verified, and there is a lack of comparative studies with single-modality neuromodulation and standard rehabilitation alone.

Compared with previous studies, this research has three key innovations: First, we expanded the sample size to 180 cases, including two additional control groups (TMS + Standard Rehabilitation and tDCS + Standard Rehabilitation) to clarify the additional effect of combined neuromodulation. Second, we extended the outcome measures to include psychiatric indicators (Beck Depression Inventory (BDI) and Generalized Anxiety Disorder 7-item scale (GAD-7)) to comprehensively evaluate post-stroke recovery. Third, we optimized the combined therapy parameters (80% resting motor threshold (RMT) for TMS and 2 mA for tDCS) based on previous evidence and consensus guidelines to enhance therapeutic efficacy [20, 21].

This study examined whether treatment with combined TMS and tDCS plus standard rehabilitation was associated with longitudinal changes in BDI, GAD-7, National Institutes of Health Stroke Scale (NIHSS), modified Rankin Scale (mRS), and Fugl-Meyer Assessment (FMA) scores compared with standard rehabilitation alone or neuromodulation with a single modality. Because treatment assignment was based on routine clinical practice rather than randomization, the analysis was intended to characterize associations rather than establish causal treatment effects. Adverse events were also compared across the four treatment groups.

## Methods

### Study Design

This retrospective cohort study was approved by the Ethics Committee of The First People’s Hospital of Linping District, Hangzhou City (Approval No.: LPYY-2024-101). All patients or their legal representatives provided written informed consent, which was documented in the medical record system. This retrospective cohort study was conducted at The First People’s Hospital of Linping District, Hangzhou City from January 2022 to December 2024. Inclusion criteria: (1) Cranial computed tomography (CT) or magnetic resonance imaging (MRI) confirmation of ischemic or hemorrhagic stroke; (2) Time since stroke onset: 1–6 months; (3) Age: 18–80 years; (4) Presence of motor dysfunction with the motor function component score of FMA < 100 (the full score of FMA motor function component is 100) [22]; (5) Stable vital signs and no contraindications to neuromodulation therapy; (6) Ability to complete follow-up assessments. Exclusion criteria: (1) History of neurological conditions like epilepsy or brain tumors; (2) Implanted electronic devices (e.g., cardiac pacemaker); (3) Severe cognitive decline, defined as an Mini-Mental State Examination (MMSE) total score <10 (range 0–30), which indicates severe cognitive impairment [23]; (4) Pregnancy or lactation; (5) Allergic to materials used in neuromodulation devices; (6) Refusal to participate or loss to follow-up; (7) Pre-existing psychiatric diagnoses (prior to stroke onset) or ongoing psychotropic medication use, to ensure that depression and anxiety outcomes measured were attributable to post-stroke emotional disturbances rather than pre-existing psychiatric conditions.

180 eligible patients were divided into four groups based on treatment records in the medical record system: the Combined Neuromodulation Group (*n* = 60, combined TMS + tDCS + standard rehabilitation), the Standard Rehabilitation Group (*n* = 60, standard rehabilitation alone), the TMS + Standard Rehabilitation Group (*n* = 30, TMS + standard rehabilitation), and the tDCS + Standard Rehabilitation Group (*n* = 30, tDCS + standard rehabilitation). It should be noted that the unequal group sizes reflect the actual distribution of patients in our clinical records, as combined neuromodulation and standard rehabilitation were prescribed far more frequently than single-modality neuromodulation treatments during the study period. All 180 enrolled patients had complete data at baseline, 4 weeks, and 8 weeks, with no attrition or missing data exclusions.

### Treatment Protocols

Standard rehabilitation (all four groups)

All patients received standard rehabilitation therapy for 5 days a week, 45 minutes per session, for 8 weeks. The program included:

(1) Physical therapy: Gait training, balance training, range of motion exercises, and strength training;

(2) Occupational therapy: ADL training, fine motor skill training, and cognitive training;

(3) Speech therapy (if needed): Language comprehension and expression training for patients with aphasia.

Neuromodulation therapy parameters

Patients in the Combined Neuromodulation Group, TMS + Standard Rehabilitation Group, and tDCS + Standard Rehabilitation Group received neuromodulation therapy in addition to standard rehabilitation, administered once daily, 5 days a week, for 8 weeks (total 40 sessions). To ensure comparability across neuromodulation groups, identical stimulation parameters were used for single-modality and combined treatment:

(1) TMS (applied in both the Combined Neuromodulation Group and the TMS + Standard Rehabilitation Group): We applied a figure-of-eight coil (Magstim Rapid2; Magstim Company Ltd., Whitland, Carmarthenshire, UK) to stimulate the M1 region opposite the affected limb. The stimulation level was fixed at 80% of the RMT. Each session included 10 trains of 50 pulses at 10 Hz, with a 1-minute inter-train interval (total 500 pulses per session).

(2) tDCS (applied in both the Combined Neuromodulation Group and the tDCS + Standard Rehabilitation Group): A DC stimulator (DC-STIMULATOR MC; NeuroConn GmbH, Ilmenau, Germany) with 5 × 7 cm electrodes was used. Anode over M1 (contralateral to the affected limb), cathode over the ipsilateral supraorbital area. Current: 2 mA; duration: 20 minutes per session.

### Outcome Measures

All outcome measures were extracted and verified by two independent rehabilitation physicians who were blinded to the treatment assignment, based on medical records.

#### Baseline Assessments

Demographic data (age, gender, stroke type, time since stroke, comorbidities), NIHSS, mRS, FMA scores (total, upper limb, lower limb, balance) and RMT (for the Combined Neuromodulation Group and the TMS + Standard Rehabilitation Group). Depression and anxiety status at baseline was determined by BDI and GAD-7 scores obtained upon hospital admission. Patients with pre-existing psychiatric diagnoses or ongoing psychotropic medications were excluded (see exclusion criterion 7).

#### Follow-Up Assessments

NIHSS, mRS, FMA, BDI, and GAD-7 scores were extracted from the medical record system at 4 weeks and 8 weeks after treatment initiation.

#### Safety Outcomes

Adverse events (e.g., headache, dizziness) were extracted from the medical record system and treatment logs, including records from treatment sessions and follow-up visits.

#### Scale Descriptions

NIHSS: Assesses neurological deficits including consciousness, language, motor function, and sensory function. It consists of 15 items, with scores ranging from 0 to 42. Higher scores indicate more severe neurological impairment. In our study population, the NIHSS demonstrated excellent inter-rater reliability (intraclass correlation coefficient (ICC) = 0.94 [95% confidence interval (CI): 0.91–0.96]) and construct validity (correlation with mRS: r = 0.78, *p* < 0.001). The scale has high inter-rater reliability (ICC = 0.92–0.96) and construct validity [24].

mRS: Evaluates functional independence and disability in daily life. It has 7 grades (0–6), with higher scores indicating greater disability (6 = death). In our population, the mRS showed good inter-rater reliability (weighted κ = 0.83 [95% CI: 0.79–0.87]) and criterion validity. It shows good reliability (κ = 0.81–0.86) and validity [25].

FMA: Assesses post-stroke motor recovery. The motor domain comprises 33 upper-limb items (0–66 points) and 17 lower-limb items (0–34 points), yielding a motor total score of 0–100; higher scores indicate better motor function. The 7-item balance subscale (0–14 points) was analyzed separately. In our study cohort, the FMA motor scores and balance subscale demonstrated excellent internal consistency (Cronbach’s α = 0.95–0.97) and test-retest reliability (ICC = 0.93–0.96). The scale has excellent reliability (ICC = 0.95–0.98) and validity [26].

BDI: Assesses depression severity. It has 21 items, with scores ranging from 0 to 63 [27]. In the present study, BDI total scores were analyzed as continuous raw scores, and no depression-severity grading was performed. In our study population, the BDI showed excellent internal consistency (Cronbach’s α = 0.91) and convergent validity with clinical diagnostic interviews (area under the curve (AUC) = 0.89, 95% CI: 0.84–0.93). Reliability: Cronbach’s α = 0.87–0.92; Validity: r = 0.78 with Diagnostic and Statistical Manual of Mental Disorders, Fourth Edition (DSM-IV) criteria.

GAD-7: Evaluates anxiety severity. It has 7 items, with scores ranging from 0 to 21. Scores <5 = no anxiety, 5–9 = mild anxiety, 10–14 = moderate anxiety, ≥15 = severe anxiety [28]. In our study population, the GAD-7 demonstrated high internal consistency (Cronbach’s α = 0.88) and diagnostic accuracy (AUC = 0.85, 95% CI: 0.79–0.90) for identifying clinically significant anxiety. Reliability: Cronbach’s α=0.89; Validity: AUC = 0.83 for diagnosing GAD [29].

### Statistical Analysis

Analyses were performed using SPSS 26.0 (IBM Corp., Armonk, NY, USA). Continuous and ordinal outcome measures (BDI, GAD-7, NIHSS, mRS, FMA motor total and subscores) were non-normally distributed or ordinal and are reported as median (25th percentile, 75th percentile). Categorical data are presented as counts (%). Baseline continuous and ordinal variables were compared among the four groups using the Kruskal-Wallis H test, because resting motor threshold (RMT) was measured in only two groups (Combined Neuromodulation Group and TMS + Standard Rehabilitation Group), the between-group difference in RMT was assessed using the Mann-Whitney U test; categorical variables were compared using the chi-square test or Fisher’s exact test when expected cell frequencies were <5. When an omnibus Kruskal-Wallis test was significant, prespecified many-to-one comparisons of each neuromodulation group with the Standard Rehabilitation Group were performed using Dunnett-adjusted pairwise tests. Generalized estimating equations (GEE) with an exchangeable working correlation structure evaluated longitudinal associations across baseline, 4 weeks, and 8 weeks. All GEE models used a Gaussian family with an identity link and robust sandwich variance estimators. Because the four treatment groups occupied ordered positions on a gradient of neuromodulation intensity, the four-level group variable was entered as an ordinal (linear) predictor coded 0 = Standard Rehabilitation, 1 = TMS + standard rehabilitation, 2 = tDCS + standard rehabilitation, and 3 = Combined (TMS + tDCS) + standard rehabilitation, so that a single group coefficient was estimated for each outcome. Time was likewise entered as a continuous linear predictor coded in weeks (0, 4, and 8). The group × time interaction was represented by the product of these two coded terms. Models were adjusted for age, gender, stroke type (ischemic vs. hemorrhagic), time since stroke onset, education level, and comorbidity burden (number of chronic conditions, including hypertension and diabetes).

These covariates were selected a priori based on their clinical relevance and availability in the medical records. Under this parameterization, the group β represents the estimated change in the outcome per one-category increase in neuromodulation intensity, the time β represents the estimated change in the outcome per week, and the group × time β represents the difference in the weekly rate of change per one-category increase in neuromodulation intensity. Because outcomes were modeled on their original scales with an identity link, all β coefficients are expressed in the units of the corresponding outcome measure (e.g., BDI points); negative β values for BDI, GAD-7, NIHSS, and mRS and positive β values for FMA indicate more favorable trajectories. A single group, time, and interaction coefficient (with corresponding Wald χ^2^ statistic and 95% CI) is therefore reported for each outcome in the text and in Tables 2–5, which ensures that the reported β coefficients and Wald statistics are presented consistently. GEE estimates are reported as regression coefficients (β), standard errors (SE), Wald χ^2^ statistics, *p* values, and 95% CI in the text; corresponding Wald statistics and *p* values are summarized in Tables 2–5. The Wilcoxon signed-rank test was used for within-group comparisons over time. Where reported, the Wilcoxon effect size was expressed as r = Z/√N. All tests were two-sided, and *p* < 0.05 was considered statistically significant.

## Results

### Baseline Characteristics of the Study Population

Data for all 180 enrolled patients were included in the final analysis; no patients were excluded because of missing data or loss to follow-up. Patients were classified into the Combined Neuromodulation Group (*n* = 60), Standard Rehabilitation Group (*n* = 60), TMS + Standard Rehabilitation Group (*n* = 30), and tDCS + Standard Rehabilitation Group (*n* = 30). No statistically significant differences were detected among the groups in the measured baseline demographics, comorbidities, or primary and secondary outcome scores (all *p* > 0.05; Table 1). The absence of statistically significant differences does not establish equivalence or exclude imbalance in unmeasured characteristics.

**Table 1.** **Baseline demographic and clinical characteristics of the four groups [M (P25, P75) or n(%)]**.

Characteristic		Combined Neuromodulation Group	Standard Rehabilitation Group	TMS + Std Rehab Group	tDCS + Std Rehab Group	Test statistic	*p* value
		(*n* = 60)	(*n* = 60)	(*n* = 30)	(*n* = 30)		
Age (years), M (P25, P75)		62.0 (55.0, 70.0)	63.5 (56.0, 71.0)	61.5 (54.0, 69.0)	64.0 (57.0, 72.0)	H = 1.03	0.789
Gender, n (%)						χ^2^ = 0.33	0.915
	Male	35 (58.3)	33 (55.0)	18 (60.0)	17 (56.7)		-
	Female	25 (41.7)	27 (45.0)	12 (40.0)	13 (43.3)		-
Stroke type, n (%)						χ^2^ = 0.65	0.876
	Ischemic	48 (80.0)	46 (76.7)	24 (80.0)	23 (76.7)		-
	Hemorrhagic	12 (20.0)	14 (23.3)	6 (20.0)	7 (23.3)		-
Time since stroke (months)		3.0 (2.0, 4.5)	3.5 (2.5, 5.0)	3.2 (2.1, 4.7)	3.3 (2.3, 4.8)	H = 1.52	0.692
Hypertension		38 (63.3)	40 (66.7)	19 (63.3)	20 (66.7)	χ^2^ = 0.61	0.894
Diabetes		22 (36.7)	20 (33.3)	11 (36.7)	10 (33.3)	χ^2^ = 0.29	0.961
BDI score		14.0 (11.0, 17.0)	13.5 (10.0, 16.5)	14.0 (10.5, 17.0)	13.5 (10.5, 16.5)	H = 0.68	0.912
GAD-7 score		9.0 (7.0, 11.0)	9.0 (7.0, 11.0)	9.0 (6.0, 11.0)	9.0 (7.0, 11.0)	H = 0.73	0.865
NIHSS		9.0 (7.0, 11.0)	8.5 (6.5, 10.5)	8.8 (7.0, 10.7)	8.7 (6.8, 10.6)	H = 0.91	0.834
mRS		3.0 (2.0, 3.0)	3.0 (2.0, 3.0)	3.0 (2.0, 3.0)	3.0 (2.0, 3.0)	H = 1.01	0.791
FMA total score		52.0 (44.0, 60.0)	52.0 (43.0, 59.0)	52.0 (44.0, 59.0)	52.0 (43.0, 59.0)	H = 0.71	0.98
RMT (% MSI), Median (P25, P75)		42.0 (38.0, 46.0)	—	41.0 (37.0, 45.0)	—	U = 1686	0.778

Abbreviations: TMS, transcranial magnetic stimulation; tDCS, transcranial direct current stimulation; Std Rehab, standard rehabilitation; BDI, Beck Depression Inventory; GAD-7, Generalized Anxiety Disorder 7-item scale; NIHSS, National Institutes of Health Stroke Scale; mRS, modified Rankin Scale; FMA, Fugl-Meyer Assessment; RMT, resting motor threshold; MSI, maximal stimulator output; M (P25, P75), median (25th percentile, 75th percentile); H, Kruskal-Wallis H statistic; χ^2^, chi-square statistic; U, Mann-Whitney U statistic. Data are expressed as median (interquartile range) or n (%). RMT was measured only in the Combined Neuromodulation Group and the TMS + Standard Rehabilitation Group and was compared using the Mann-Whitney U test. No statistically significant differences were detected in the measured baseline characteristics.

### Changes in BDI and GAD-7 Scores Over Time

GEE analysis identified significant group × time interactions for both BDI (β = −1.85, SE = 0.24, Wald χ^2^ = 58.24, *p* < 0.001, 95% CI: −2.32–−1.38) and GAD-7 (β = −1.52, SE = 0.22, Wald χ^2^ = 45.67, *p* < 0.001, 95% CI: −1.95–−1.09). The main effects of group were also significant (BDI: β = −4.52, SE = 0.42, Wald χ^2^ = 112.35, *p* < 0.001, 95% CI: −5.34–−3.70; GAD-7: β = −3.18, SE = 0.34, Wald χ^2^ = 89.64, *p* < 0.001, 95% CI: −3.85–−2.51), as were the main effects of time (BDI: β = −3.28, SE = 0.26, Wald χ^2^ = 156.78, *p* < 0.001, 95% CI: −3.79–−2.77; GAD-7: β = −2.24, SE = 0.19, Wald χ^2^ = 134.52, *p* < 0.001, 95% CI: −2.61–−1.87).

At 4 and 8 weeks, the Combined Neuromodulation Group had the lowest median BDI and GAD-7 scores. In the prespecified Dunnett-adjusted comparisons with the Standard Rehabilitation Group, the combined, TMS, and tDCS groups each had lower BDI and GAD-7 scores at both follow-up assessments (Table 2). These many-to-one comparisons did not test direct differences among the three neuromodulation groups.

**Table 2.** **Changes in BDI and GAD-7 scores over time in the four groups [M (P25, P75)]**.

Outcome measure	Time point	Combined neuromodulation	Standard rehabilitation	TMS + Std Rehab	tDCS + Std Rehab	Group effect	Time effect	Interaction Wald	Pairwise comparison *p* value
		Group (*n* = 60)	Group (*n* = 60)	Group (*n* = 30)	Group (*n* = 30)	Wald χ^2^ (*p*)	Wald χ^2^ (*p*)	χ^2^ (*p*)	(Dunnett-adjusted vs. Std)
BDI score	Baseline	14.0 (11.0, 17.0)	13.5 (10.0, 16.5)	14.0 (10.5, 17.0)	13.5 (10.5, 16.5)	112.35 (<0.001)	156.78 (<0.001)	58.24 (<0.001)	All *p * > 0.05
	4 weeks	10.0 (8.0, 13.0)	16.0 (13.0, 19.0)	13.5 (11.0, 16.0)	14.0 (11.5, 16.5)				Combined vs. Std: <0.001; TMS vs. Std: <0.001; tDCS vs. Std: 0.002
	8 weeks	8.0 (6.0, 10.0)	14.5 (11.5, 17.0)	11.5 (9.0, 14.0)	12.0 (9.5, 14.5)				Combined vs. Std: <0.001; TMS vs. Std: <0.001; tDCS vs. Std: <0.001
GAD-7 score	Baseline	9.0 (7.0, 11.0)	9.0 (7.0, 11.0)	9.0 (6.0, 11.0)	9.0 (7.0, 11.0)	89.64 (<0.001)	134.52 (<0.001)	45.67 (<0.001)	All *p * > 0.05
	4 weeks	7.0 (5.0, 9.0)	10.0 (8.0, 12.0)	8.5 (6.0, 10.0)	9.0 (7.0, 11.0)				Combined vs. Std: <0.001; TMS vs. Std: <0.001; tDCS vs. Std: <0.001
	8 weeks	6.0 (4.0, 8.0)	9.0 (7.0, 11.0)	8.0 (6.0, 10.0)	8.0 (6.0, 10.0)				Combined vs. Std: <0.001; TMS vs. Std: <0.001; tDCS vs. Std: <0.001

Abbreviations: BDI, Beck Depression Inventory; GAD-7, Generalized Anxiety Disorder 7-item scale; Std, standard rehabilitation; TMS, transcranial magnetic stimulation; tDCS, transcranial direct current stimulation; M (P25, P75), median (25th percentile, 75th percentile). Data are expressed as median (interquartile range). Wald χ^2^ and *p* values for the group, time, and group × time terms were derived from generalized estimating equation models adjusted for age, gender, stroke type, time since stroke onset, education level, and comorbidity burden. Pairwise P values are Dunnett-adjusted comparisons with the Standard Rehabilitation Group as reference.

### Changes in NIHSS Scores Over Time

NIHSS scores decreased over time in all four groups. At both follow-up assessments, each neuromodulation group had lower NIHSS scores than the Standard Rehabilitation Group (all Dunnett-adjusted *p* < 0.001), and the Combined Neuromodulation Group had the lowest median scores. GEE analysis identified a significant group × time interaction (β = −1.28, SE = 0.20, Wald χ^2^ = 42.36, *p* < 0.001, 95% CI: −1.67–−0.89), together with significant main effects of group (β = −2.85, SE = 0.31, Wald χ^2^ = 86.52, *p* < 0.001, 95% CI: −3.46–−2.24) and time (β = −1.96, SE = 0.18, Wald χ^2^ = 118.43, *p* < 0.001, 95% CI: −2.31–−1.61) (Table 3).

**Table 3.** **Median (IQR) NIHSS score changes over time [M (P25, P75)]**.

Time point	Combined neuromodulation	Standard rehabilitation	TMS + Std Rehab	tDCS + Std Rehab	Group Wald	Time Wald	Group × time Wald	Pairwise comparison *p* value
	Group (*n* = 60)	Group (*n* = 60)	Group (*n* = 30)	Group (*n* = 30)	χ^2^ (*p*)	χ^2^ (*p*)	χ^2^ (*p*)	(Dunnett-adjusted vs. Std)
Baseline	9.0 (7.0, 11.0)	8.5 (6.5, 10.5)	8.8 (7.0, 10.7)	8.7 (6.8, 10.6)	86.52 (<0.001)	118.43 (<0.001)	42.36 (<0.001)	All *p * > 0.05
4 weeks	5.0 (3.0, 7.0)	7.5 (5.0, 9.0)	6 (4.0, 8.0)	6.2 (4.2, 8.1)				Combined vs. Std: <0.001; TMS vs. Std: <0.001; tDCS vs. Std: <0.001
8 weeks	3.0 (2.0, 5.0)	6.0 (4.0, 8.0)	4.5 (3.0, 6.5)	4.8 (3.2, 6.8)				Combined vs. Std: <0.001; TMS vs. Std: <0.001; tDCS vs. Std: <0.001

Abbreviations: NIHSS, National Institutes of Health Stroke Scale; Std, standard rehabilitation; TMS, transcranial magnetic stimulation; tDCS, transcranial direct current stimulation; M (P25, P75), median (25th percentile, 75th percentile); IQR, interquartile range. Data are expressed as median (interquartile range). Wald χ^2^ and *p* values were derived from generalized estimating equation models adjusted for prespecified covariates. Pairwise *p* values are Dunnett-adjusted comparisons with the Standard Rehabilitation Group as reference.

### Changes in mRS Scores Over Time

mRS scores decreased over time in all four groups. At 4 and 8 weeks, each neuromodulation group had lower mRS scores than the Standard Rehabilitation Group in Dunnett-adjusted comparisons (all *p* < 0.05). GEE analysis identified a significant group × time interaction (β = −0.48, SE = 0.08, Wald χ^2^ = 35.72, *p* < 0.001, 95% CI: −0.64–−0.32), as well as significant main effects of group (β = −0.85, SE = 0.10, Wald χ^2^ = 72.18, *p* < 0.001, 95% CI: −1.05–−0.65) and time (β = −0.62, SE = 0.06, Wald χ^2^ = 98.65, *p* < 0.001, 95% CI: −0.74–−0.50). Because mRS is an ordinal scale, values are reported as medians (Table 4).

**Table 4.** **Median (IQR) mRS scores changes over time [M (P25, P75)]**.

Time point	Combined neuromodulation	Std Rehab	TMS + Std Rehab	tDCS + Std Rehab	Group Wald	Time Wald	Group × time Wald	Pairwise comparison *p*
	Group (*n* = 60)	Group (*n* = 60)	Group (*n* = 30)	Group (*n* = 30)	χ^2^ (*p*)	χ^2^ (*p*)	χ^2^ (*p*)	(Dunnett-adjusted vs. Std)
Baseline	3.0 (2.0, 3.0)	3.0 (2.0, 3.0)	3.0 (2.0, 3.0)	3.0 (2.0, 3.0)	72.18 (<0.001)	98.65 (<0.001)	35.72 (<0.001)	All *p * > 0.05
4 weeks	2.0 (1.0, 2.0)	2.0 (1.5, 3.0)	2.0 (1.5, 2.5)	2.0 (1.5, 2.5)				Combined vs. Std: <0.001; TMS vs. Std: 0.038; tDCS vs. Std: 0.026
8 weeks	1.0 (0.0, 1.0)	2.0 (1.0, 2.5)	1.0 (1.0, 2.0)	1.0 (1.0, 2.0)				Combined vs. Std: <0.001; TMS vs. Std: 0.042; tDCS vs. Std: 0.031

Abbreviations: mRS, modified Rankin Scale; Std, standard rehabilitation; TMS, transcranial magnetic stimulation; tDCS, transcranial direct current stimulation; M(P25, P75), median (25th percentile, 75th percentile); IQR, interquartile range. Wald χ^2^ and *p* values were derived from generalized estimating equation models adjusted for prespecified covariates. Pairwise *p* values are Dunnett-adjusted comparisons with the Standard Rehabilitation Group as reference. mRS is an ordinal scale (0–6); results are reported as medians. Test statistics (group, time, interaction effects) derived from GEE with Wald χ^2^.

### Changes in FMA Scores (Total and Subscores) Over Time

A ceiling effect was suggested for the FMA lower-limb and balance scores in the Combined Neuromodulation Group at 8 weeks [lower limb: 32.0 (30.0, 34.0); balance: 13.0 (12.0, 14.0)]. Interpretation of these subscores should therefore consider their limited discrimination among patients approaching the upper end of the scale.

FMA motor total, upper-limb, lower-limb, and balance scores increased over time in all four groups (all Wilcoxon *p* < 0.001; r = 0.68−0.78). In Dunnett-adjusted comparisons, each neuromodulation group had higher FMA scores than the Standard Rehabilitation Group at both follow-up assessments (all *p* < 0.05). GEE analyses identified significant group, time, and group × time terms for all FMA outcomes (Table 5).

**Table 5.** **Changes in FMA scores in the four groups at different time points [M(P25, P75)]**.

Outcome measure	Time point	Combined neuromodulation	Std Rehab group	TMS + Std Rehab	tDCS + Std Rehab	Overall group	Overall time	Group × time	Pairwise comparison *p*
		Group (*n* = 60)	(*n* = 60)	Group (*n* = 30)	Group (*n* = 30)	*p* (Wald χ^2^)	*p* (Wald χ^2^)	Interaction *p*	(Dunnett-adjusted vs. Std)
FMA total score	Baseline	52.0 (44.0, 60.0)	52.0 (43.0, 59.0)	52.0 (44.0, 59.0)	52.0 (43.0, 59.0)	All *p * < 0.001	All *p * < 0.001	All *p * < 0.001	All *p * > 0.05
	4 weeks	68.0 (60.0, 75.0)	60.0 (52.0, 67.0)	63.5 (56.0, 70.0)	63.0 (55.0, 69.0)				Combined vs. Std: <0.001; TMS vs. Std: <0.001; tDCS vs. Std: <0.001
	8 weeks	83.0 (76.0, 89.0)	70.0 (63.0, 77.0)	75.0 (68.0, 82.0)	74.5 (67.0, 81.0)				Combined vs. Std: <0.001; TMS vs. Std: 0.012; tDCS vs. Std: 0.010
FMA upper limb	Baseline	24.0 (18.0, 30.0)	23.0 (17.0, 29.0)	24.0 (17.0, 29.0)	23.0 (17.0, 28.0)				All *p * > 0.05
	4 weeks	45.0 (38.0, 52.0)	32.0 (26.0, 38.0)	38.0 (31.0, 44.0)	37.0 (30.0, 43.0)				Combined vs. Std: <0.001; TMS vs. Std: 0.008; tDCS vs. Std: 0.015
	8 weeks	52.0 (46.0, 58.0)	38.0 (32.0, 44.0)	45.0 (39.0, 51.0)	44.0 (38.0, 50.0)				Combined vs. Std: <0.001; TMS vs. Std: <0.05; tDCS vs. Std: <0.05
FMA lower limb	Baseline	24.0 (20.0, 28.0)	23.5 (20.0, 27.0)	24.0 (20.0, 27.0)	23.5 (20.0, 27.0)				All *p * > 0.05
	4 weeks	30.0 (27.0, 32.0)	28.0 (25.0, 30.0)	29.0 (26.0, 31.0)	28.5 (26.0, 31.0)				Combined vs. Std: <0.001; TMS vs. Std: 0.032; tDCS vs. Std: 0.021
	8 weeks	32.0 (30.0, 34.0)	30.0 (27.0, 32.0)	31.0 (28.0, 33.0)	31.0 (28.0, 33.0)				Combined vs. Std: 0.003; TMS vs. Std: 0.047; tDCS vs. Std: 0.038
FMA balance	Baseline	8.0 (7.0, 10.0)	8.0 (7.0, 10.0)	8.0 (7.0, 10.0)	8.0 (7.0, 10.0)				All *p * > 0.05
	4 weeks	11.0 (10.0, 12.0)	10.0 (9.0, 11.0)	10.5 (9.5, 12.0)	10.5 (9.5, 11.5)				Combined vs. Std: <0.001; TMS vs. Std: 0.028; tDCS vs. Std: 0.035
	8 weeks	13.0 (12.0, 14.0)	11.0 (10.0, 13.0)	12.0 (11.0, 13.0)	12.0 (11.0, 13.0)				Combined vs. Std: <0.001; TMS vs. Std: 0.015; tDCS vs. Std: 0.020

Abbreviations: FMA, Fugl-Meyer Assessment; Std, standard rehabilitation; TMS, transcranial magnetic stimulation; tDCS, transcranial direct current stimulation; M(P25, P75), median (25th percentile, 75th percentile); IQR, interquartile range. Data are expressed as median (interquartile range). Wald χ^2^ and *p* values were derived from generalized estimating equation models adjusted for prespecified covariates. Pairwise *p* values are Dunnett-adjusted comparisons with the Standard Rehabilitation Group as reference. The between-group comparisons for FMA outcomes are presented as Dunnett-adjusted *p* values in Table 5.

For FMA motor total, the group effect was β = 12.84 (SE = 1.18, Wald χ^2^ = 118.73, *p* < 0.001, 95% CI: 10.53−15.15), the time effect was β = 15.67 (SE = 1.02, Wald χ^2^ = 236.45, *p* < 0.001, 95% CI: 13.67−17.67), and the group × time interaction was β = 3.85 (SE = 0.68, Wald χ^2^ = 31.92, *p* < 0.001, 95% CI: 2.52−5.18).

For FMA upper limb, the corresponding estimates were β = 7.42 (SE = 0.95, Wald χ^2^ = 60.89, *p* < 0.001, 95% CI: 5.56−9.28), β = 8.36 (SE = 0.82, Wald χ^2^ = 103.47, *p* < 0.001, 95% CI: 6.75−9.97), and β = 2.15 (SE = 0.54, Wald χ^2^ = 15.84, *p* < 0.001, 95% CI: 1.09−3.21).

For FMA lower limb, the estimates were β = 2.18 (SE = 0.52, Wald χ^2^ = 17.58, *p* < 0.001, 95% CI: 1.16−3.20), β = 3.24 (SE = 0.48, Wald χ^2^ = 45.56, *p* < 0.001, 95% CI: 2.30−4.18), and β = 0.72 (SE = 0.31, Wald χ^2^ = 5.42, *p* = 0.020, 95% CI: 0.11−1.33).

For FMA balance, the estimates were β = 1.35 (SE = 0.38, Wald χ^2^ = 12.68, *p* < 0.001, 95% CI: 0.61−2.09), β = 2.16 (SE = 0.35, Wald χ^2^ = 38.01, *p* < 0.001, 95% CI: 1.48−2.84), and β = 0.56 (SE = 0.24, Wald χ^2^ = 5.44, *p* = 0.020, 95% CI: 0.09−1.03), respectively.

### Safety Outcomes

Across all groups, no severe adverse events were reported during the whole treatment period. Mild adverse events were self-limiting or resolvable with simple intervention. Mild headache occurred in 3.3%−5.0% of patients, and mild skin redness at tDCS sites was observed in 2 patients (3.3%) in the Combined Neuromodulation Group, resolving after adjusting electrode placement. Given that several cells in the contingency table had frequencies less than 5, Fisher’s exact test was used rather than the Chi-square test to compare adverse event incidence. The incidence of adverse events did not vary significantly by group (*p* = 0.584) (Table 6).

**Table 6.** **Incidence of adverse events in the four groups [n(%)]**.

Adverse event type	Combined neuromodulation	Std Rehab group	TMS + Std Rehab	tDCS + Std Rehab	*p* value
	Group (*n* = 60)	(*n* = 60)	Group (*n* = 30)	Group (*n* = 30)	(fisher’s exact test)
Mild headache	3 (5.0)	2 (3.3)	1 (3.3)	1 (3.3)	0.782
Skin redness	2 (3.3)	0 (0.0)	0 (0.0)	0 (0.0)	0.245
Dizziness	0 (0.0)	1 (1.7)	0 (0.0)	0 (0.0)	0.467
Total adverse events	5 (8.3)	3 (5.0)	1 (3.3)	1 (3.3)	0.584

Abbreviations: Std Rehab, standard rehabilitation; TMS, transcranial magnetic stimulation; tDCS, transcranial direct current stimulation; n, number of patients. Data are presented as n (%). *p* values were derived from Fisher’s exact test because several expected cell frequencies were <5.

## Discussion

This retrospective cohort study compared longitudinal psychological, neurological, and motor outcomes across four treatment patterns over 8 weeks. The Combined Neuromodulation Group had the lowest median BDI and GAD-7 scores at follow-up, and the adjusted GEE models identified significant group × time associations. More favorable NIHSS, mRS, and FMA trajectories were also observed in the neuromodulation groups compared with standard rehabilitation. Recorded adverse-event rates did not differ significantly among groups. These findings describe associations and should not be interpreted as proof of treatment efficacy or causality.

The associations observed for BDI and GAD-7 are biologically plausible, but the present data cannot establish a mechanism. Both 10-Hz TMS and anodal tDCS were applied to the M1 contralateral to the affected limb (the ipsilesional M1) and may modulate cortical excitability and network plasticity [30, 31, 32]. Emotional recovery after stroke involves distributed limbic, prefrontal, and motor networks, so modulation of sensorimotor circuits could be related to psychological outcomes. This explanation remains hypothetical because cortical excitability and network connectivity were not measured.

Preclinical and neurophysiological studies have linked non-invasive brain stimulation to changes in BDNF expression, synaptic plasticity, and motor-network organization [33, 34]. These findings provide context for the observed associations but cannot be extrapolated as the molecular basis of the present results. The study did not measure BDNF or other molecular markers, and the contributions of spontaneous recovery, rehabilitation exposure, and nonspecific treatment effects cannot be separated.

Prior research has reported favorable motor outcomes after combined or dual neuromodulation strategies [35]. The current study extends this evidence by including psychological outcomes and three assessment time points in a larger clinical cohort. Nevertheless, differences in design, stimulation targets, treatment allocation, and outcome definitions limit direct comparison with earlier trials.

At 4 and 8 weeks, each neuromodulation group was compared with the Standard Rehabilitation Group using Dunnett-adjusted tests. The combined group had the most favorable median values for several outcomes; however, these many-to-one comparisons do not establish superiority over the single-modality groups. In addition, the study was not designed as a factorial trial and did not test a TMS × tDCS interaction. The data therefore cannot confirm a synergistic effect.

The lower BDI and GAD-7 scores observed in the Combined Neuromodulation Group may be clinically relevant because post-stroke depression and anxiety are associated with poorer rehabilitation participation and quality of life [36, 37]. Psychological and physical recovery may influence each other, but their temporal direction cannot be determined from the present analyses. Changes in motor function could affect mood, mood could affect rehabilitation engagement, and both may reflect shared recovery-related factors.

These observations support further evaluation of combined neuromodulation as an adjunct to standard rehabilitation rather than its immediate adoption as an established superior treatment. Future studies should prospectively document treatment adherence, concomitant therapies, changes in psychotropic medication, and rehabilitation dose, and should use randomized allocation when feasible.

The absence of neurophysiological measurements is especially relevant to mechanistic interpretation. Multimodal neuroimaging, motor-evoked potentials, and molecular markers could help determine whether changes in cortical excitability or network connectivity mediate psychological and motor outcomes. Until such evidence is available, mechanistic explanations should be regarded as hypotheses.

No severe adverse events were documented, and overall recorded adverse-event incidence did not differ among the four groups (*p* = 0.584). The stimulation parameters were within ranges commonly used in clinical research [38, 39]. However, retrospective ascertainment may miss mild or delayed events, and the sample was not powered to exclude uncommon harms. The findings therefore indicate no detected safety signal during the 8-week observation period rather than establish safety.

This study has several limitations. Treatment assignment was not randomized and reflected physician recommendations and patient preference, creating selection and indication bias. Although the GEE models adjusted for age, gender, stroke type, time since stroke, education level, and comorbidity burden, lesion location and volume, baseline motivation, socioeconomic status, rehabilitation adherence and intensity, family support, and other concomitant treatments were not fully captured. Residual and unmeasured confounding may therefore explain part of the observed associations. The absence of statistically significant differences in measured baseline variables does not establish equivalence among groups. Unequal group sizes may also reduce precision, particularly for the single-modality groups. The analysis was limited to the 180 patients with complete data at baseline, 4 weeks, and 8 weeks; this complete-case restriction may have introduced selection bias and reduced the representativeness of the analytic cohort. Although robust variance estimators were used, the sensitivity of the GEE estimates to alternative distribution families, link functions, and working correlation structures was not formally assessed. Follow-up was limited to 8 weeks, cognitive function and quality of life were not assessed, and the single-center setting limits generalizability.

Accordingly, the results should be considered hypothesis-generating. Multicenter prospective studies with adequate sample sizes, prespecified confounder control, direct comparisons among neuromodulation regimens, and longer follow-up are needed to determine whether combined TMS and tDCS confers additional clinical benefit.

## Conclusions

Among patients in this retrospective cohort, combined TMS and tDCS plus standard rehabilitation was associated with lower depression and anxiety symptom scores and more favorable neurological and motor outcome trajectories than standard rehabilitation over 8 weeks. No between-group difference in recorded adverse events was detected. Because treatment allocation was non-random and residual confounding remained, these findings do not establish causality, superiority over single-modality neuromodulation, or a synergistic mechanism. Prospective randomized studies are needed to confirm these associations.

## Availability of Data and Materials

All the data of this study are included in this article. The datasets used and/or analyzed during the current study are available from the corresponding author on reasonable request.
